# Supplementary material for: Thioporidiols A and B: Two New Sulfur Compounds Discovered by Molybdenum-Catalyzed Oxidation Screening from Trichoderma polypori FKI-7382
Source: Antibiotics (Basel). 2020 May 7;9(5):236. doi: 10.3390/antibiotics9050236 (PMC7277456; doi:10.3390/antibiotics9050236)
Supplement: Supplementary file 1 [file antibiotics-09-00236-s001.pdf]

Thioporidiols A and B: Two new sulfur compounds discovered by molybdenum-catalyzed oxidation screening from *Trichoderma polypori* FKI-7382

Hirotaka Matsuo,[*,1,2] Yoshihiko Noguchi,[1,2] Rei Miyano,[1] Mayuka Higo,[1] Kenichi Nonaka,[1,2] Toshiaki Sunazuka,[1,2] Yōko Takahashi,[1] Satoshi Ōmura,[1] and Takuji Nakashima[*,1,2]

[†]Kitasato Institute for Life Sciences, Kitasato University, Tokyo 108-8641, Japan

[‡]Graduate School of Infection Control Sciences, Kitasato University, Tokyo 108-8641, Japan

*Corresponding author. Tel and Fax: +81 3 5791 6450

E-mail address: matsu-h@lisci.kitasato-u.ac.jp (H. Matsuo), takuji@lisci.kitasato-u.ac.jp (T. Nakashima).

List of supplementary contents

- Methods of anti-cancer and anti-malarial activities.
- Figure S1 ^{1}H NMR (500 MHz, CD_3OD) spectrum of **1**.
- Figure S2 ^{13}C NMR (125 MHz, CD_3OD) spectrum of **1**.
- Figure S3 ^{1}H–^{1}H COSY (CD_3OD) spectrum of **1**.
- Figure S4 HMQC (CD_3OD) spectrum of **1**.
- Figure S5 HMBC (CD_3OD) spectrum of **1**.
- Figure S6 ROESY (CD_3OD) spectrum of **1**.
- Figure S7 ^{1}H NMR (500 MHz, CD_3OD) spectrum of **2**.
- Figure S8 ^{13}C NMR (125 MHz, CD_3OD) spectrum of **2**.
- Figure S9 ^{1}H–^{1}H COSY (CD_3OD) spectrum of **2**.
- Figure S10 HMQC (CD_3OD) spectrum of **2**.
- Figure S11 HMBC (CD_3OD) spectrum of **2**.
- Figure S12 ROESY (CD_3OD) spectrum of **2**.
- Figure S13 ^{1}H NMR (500 MHz, $CDCl_3$) spectrum of **4** measured at 25°C and the structure of MPA.
- Figure S14 ^{1}H NMR (500 MHz, $CDCl_3$) spectrum of **4** measured at –30°C.

Methods of anti-cancer and anti-malarial activities

*Cytotoxic assay of **1** and **2***

Cytotoxic activity of **1** and **2** were measured using WST-8 (Kishida Chemical, Osaka, Japan) against eight cell lines, namely HL-60 (human promyelocytic leukemia cells), Jurkat (human acute lymphocytic leukemia cell line), THP-1 (human acute monocytic leukemia cell line), HeLa S3 (human cervical cancer cell line), A549 (Human lung carcinoma cell line), Panc1 (human pancreas carcinoma cell line), HT29 (human colon adenocarcinoma cell line), and H1299 (human non-small lung carcinoma cell line). Briefly, HL-60, Jurkat, and THP-1 cell lines were seeded in 96-well plates (3×10^5 cells per well) and cultured in Roswell Park Memorial Institute (RPMI) 1640 medium (Nacalai Tesque, Kyoto, Japan) supplemented with 10% fetal bovine serum (FBS), 100 IU/mL penicillin and 100 µg/mL streptomycin (Life Technologies Japan, Tokyo, Japan) and 1 mM sodium pyruvate (Life Technologies, Tokyo, Japan). HeLa S3, A549, Panc1, HT29, and H1299 cell lines were seeded in 96-well plates (5×10^3 cells per well) and cultured in Dulbecco's modified Eagle's medium (Wako Pure Chemical Industries, Osaka, Japan) supplemented with 10% FBS and 100 IU/mL penicillin and 100 µg/mL streptomycin at 37 °C under 5% CO_2. After overnight cultured cells, **1** and **2** dissolved in MeOH at final concentrations (1.0, 3.0, 10, 30, and 100 µM) were added into each well. After 48 h of incubation at 37 °C, WST-8 solution was added to each well and incubated at 37°C for 4 h. The absorbance at 450 nm of each well was measured using a Corona Grating Microplate Reader SH-9000 (Corona Electric, Ibaraki, Japan).

*Anti-malarial activity of **1** and **2***

The evaluation of antimalarial activity was conducted as previously reported [1]. Briefly, cultured *P. falciparum* (chloroquine sensitive FCR3 strain and chloroquine resistant K1 strain) in Type A+ blood was seeded in 96-well culture plates (parasitaemia 0.5-1%, Hematocrit 2.0%) and incubated with test compounds (final concentration at 4.38, 8.75, 17.5, 35, 70 µM) for 72 h in RPMI medium supplemented with 10% human plasma at 37 ºC, under 93% N_2, 4% CO2, and 3% O_2. After incubation, parasite lactate dehydrogenase

activity was assayed to determine parasite growth and calculate the antimalarial activity in comparison with the controls that had received no compounds. This study was approved by “Kitasato Institute Hospital Research Ethics Committee (No12102)” on the donation of human erythrocytes from volunteers.

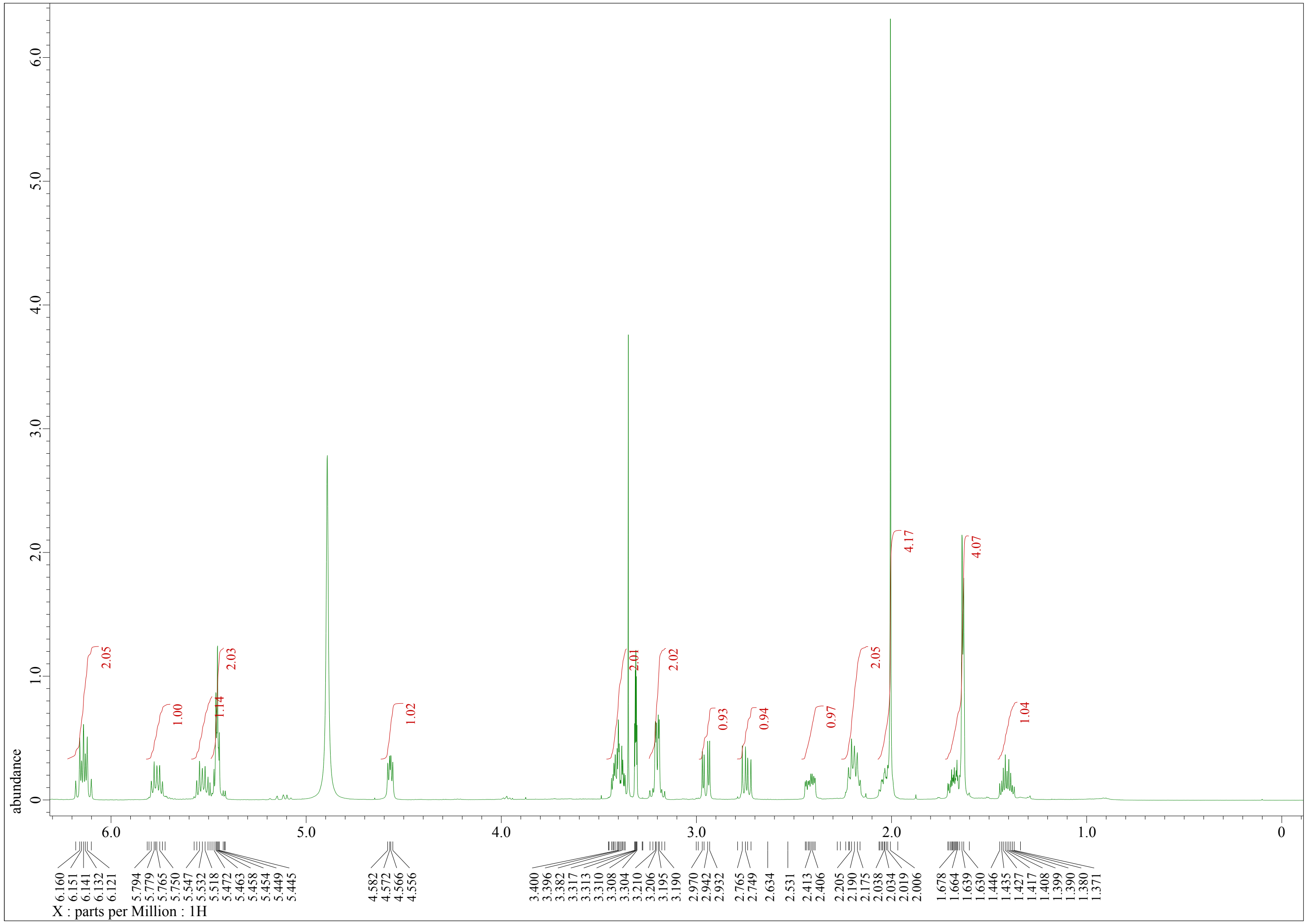

Figure S1 ^{1}H NMR (500 MHz, CD_3OD) spectrum of **1**

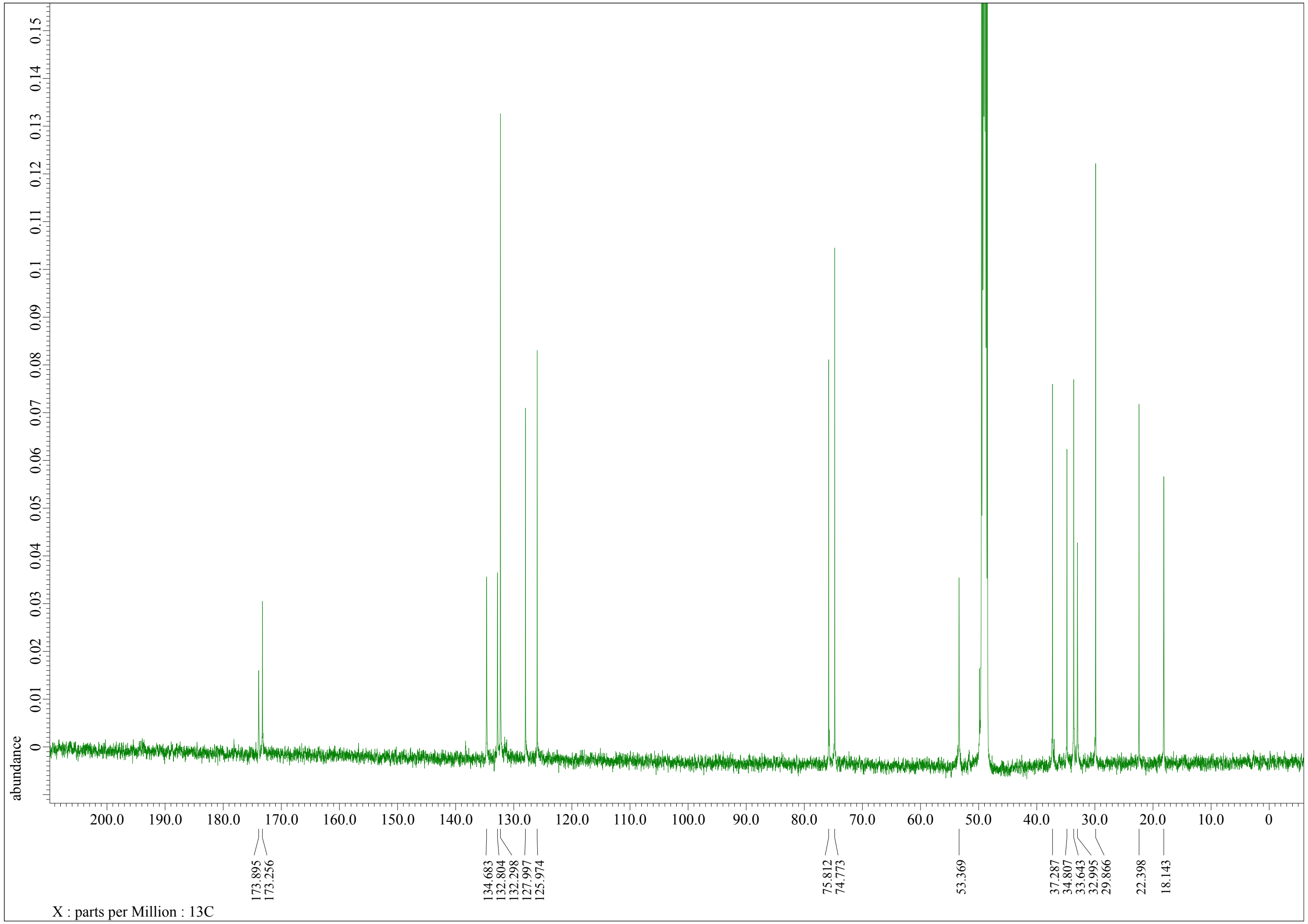

Figure S2 ^{13}C NMR (125 MHz, CD_3OD) spectrum of **1**

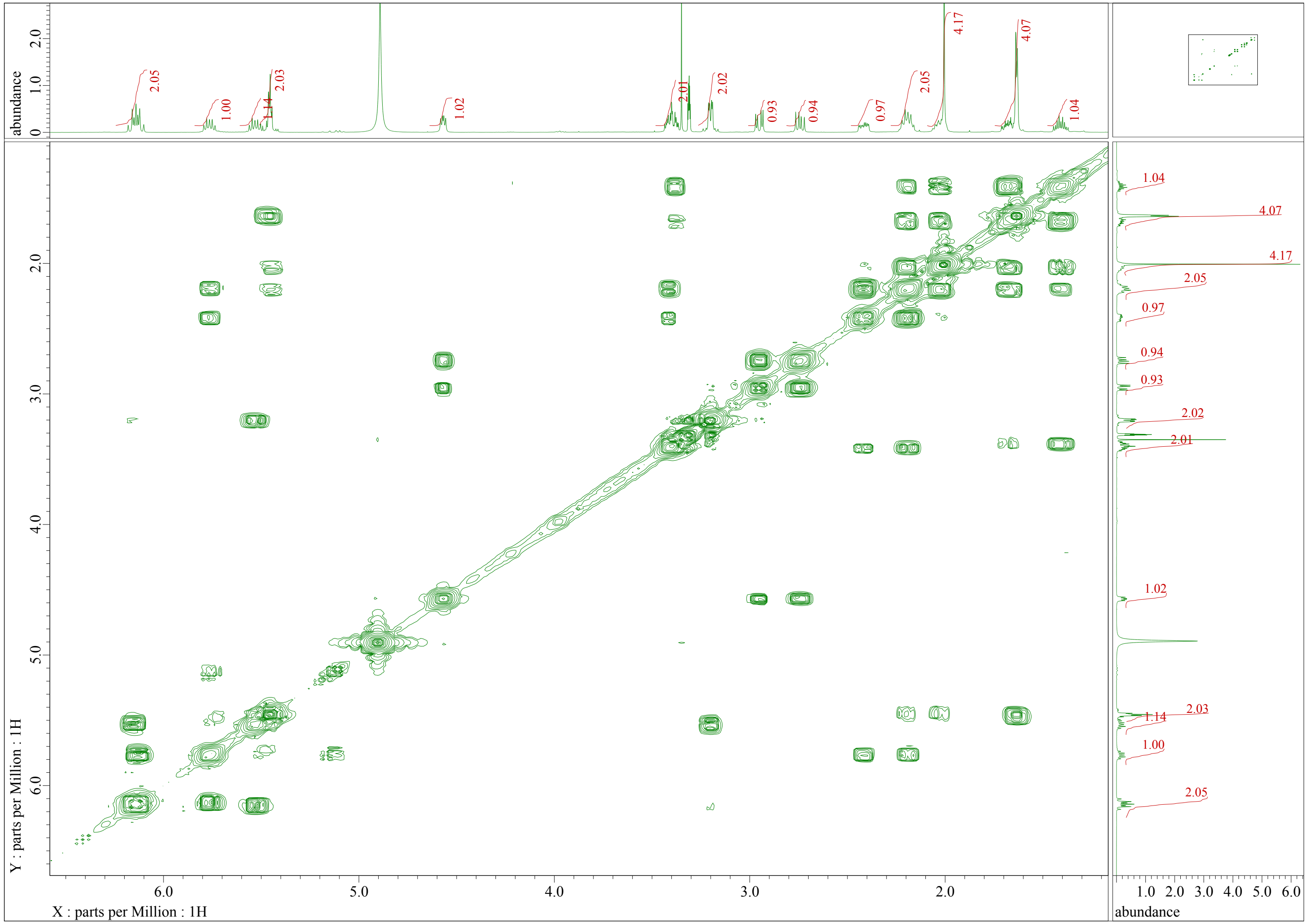

Figure S3 ^{1}H-^{1}H COSY (CD_3OD) spectrum of **1**

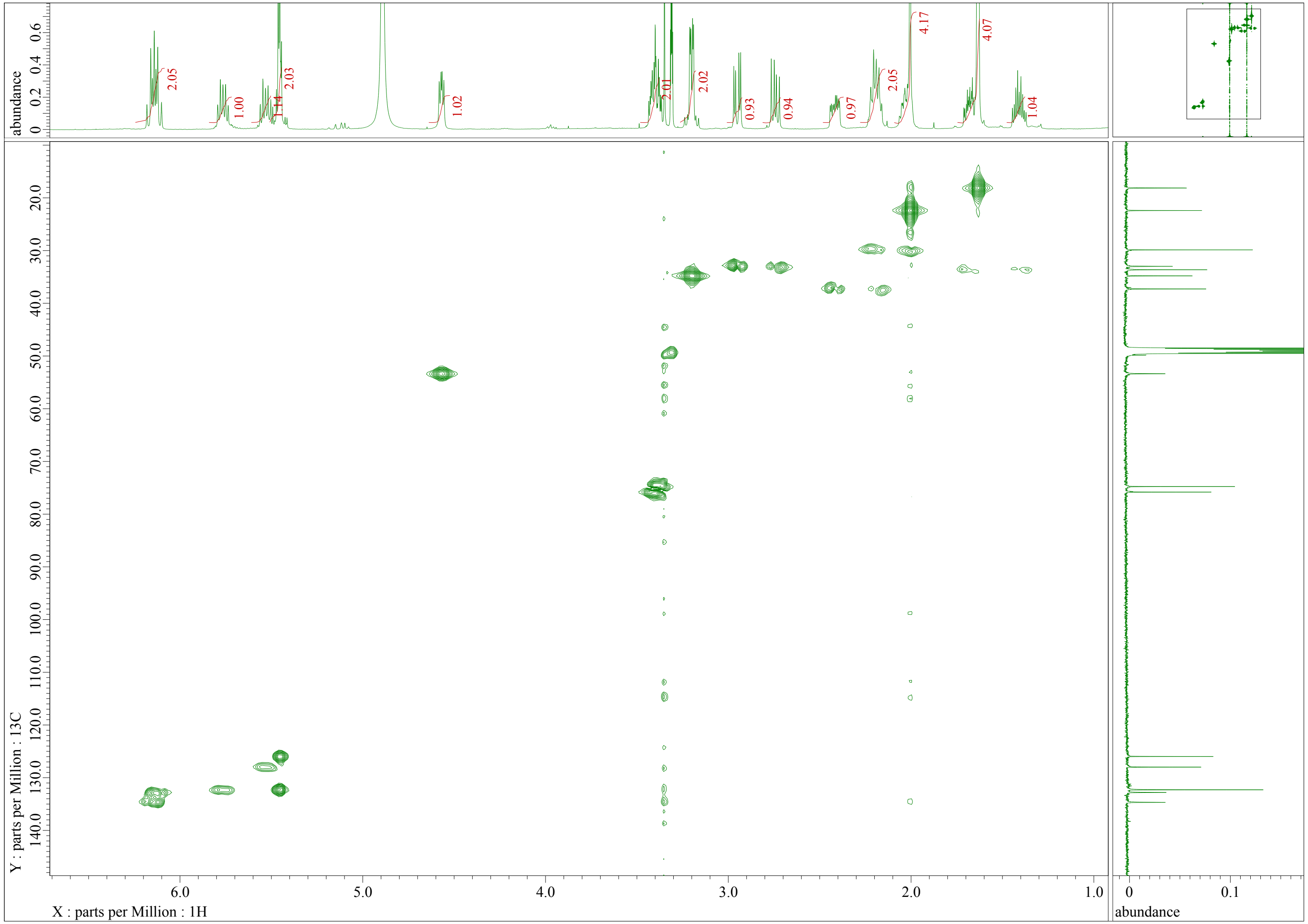

Figure S4 HMQC (CD_3OD) spectrum of **1**

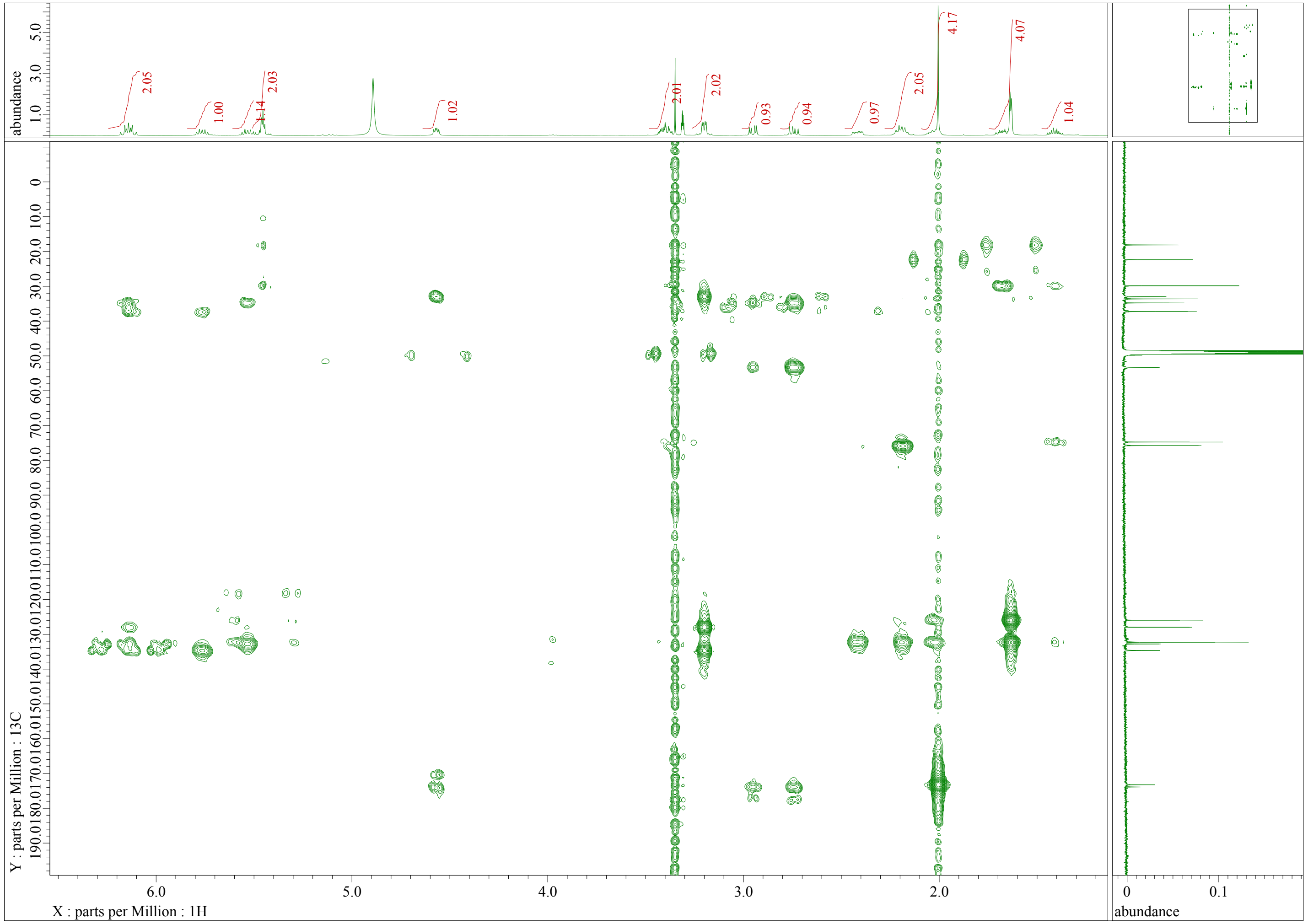

Figure S5 HMBC (CD_3OD) spectrum of **1**

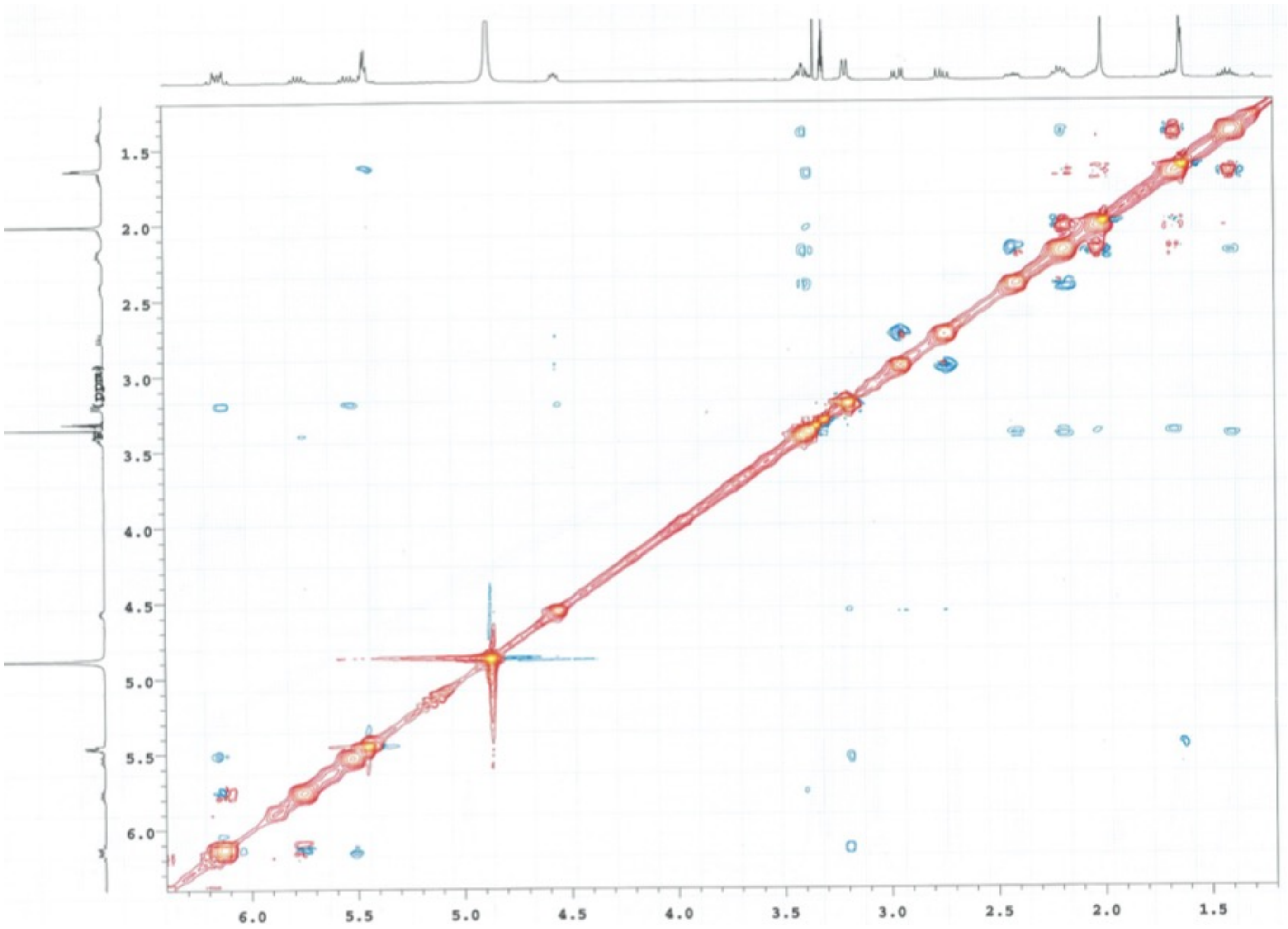

Figure S6 ROESY (CD_3OD) spectrum of **1**

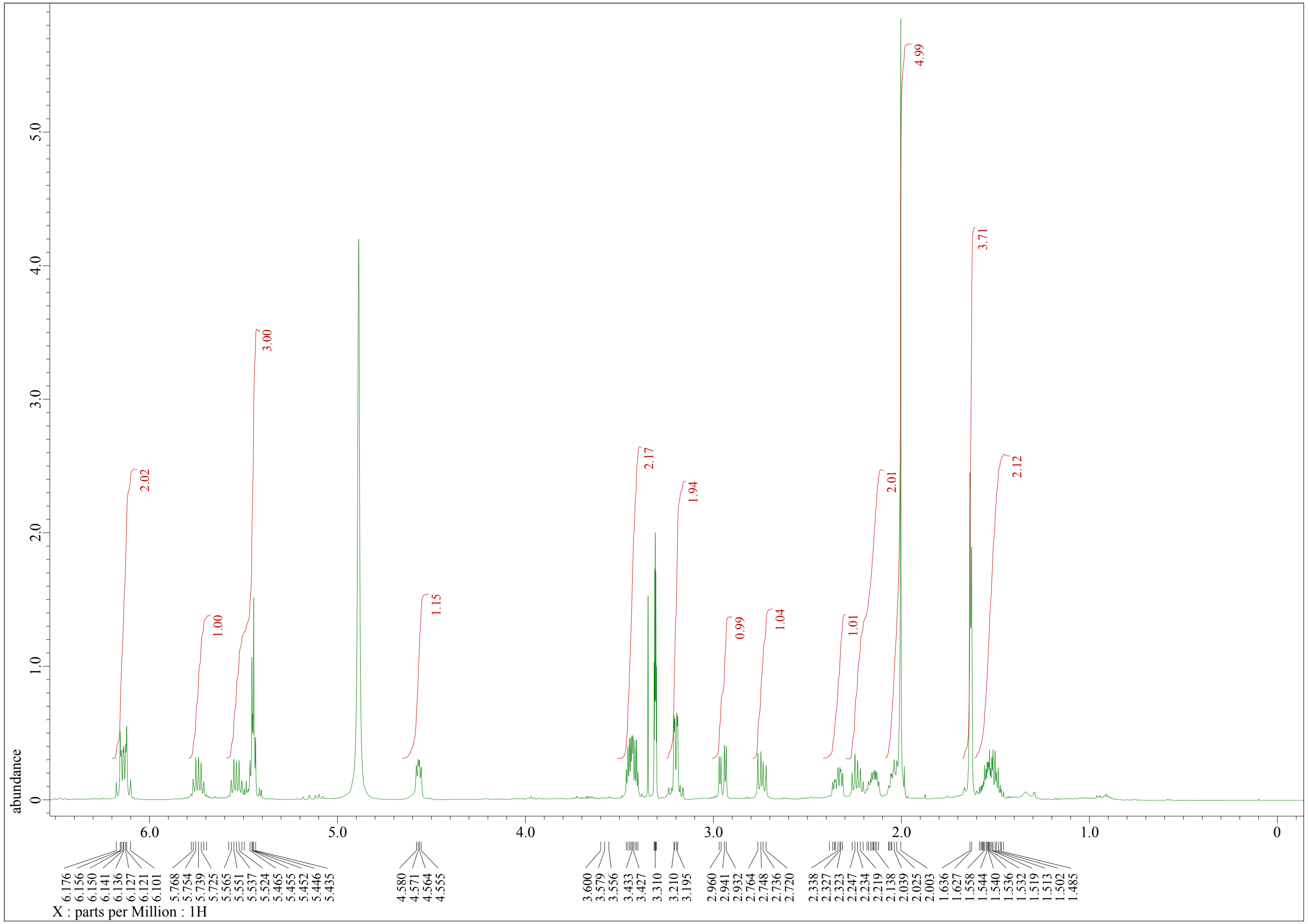

Figure S7 ^{1}H NMR (500 MHz, CD_3OD) spectrum of **2**

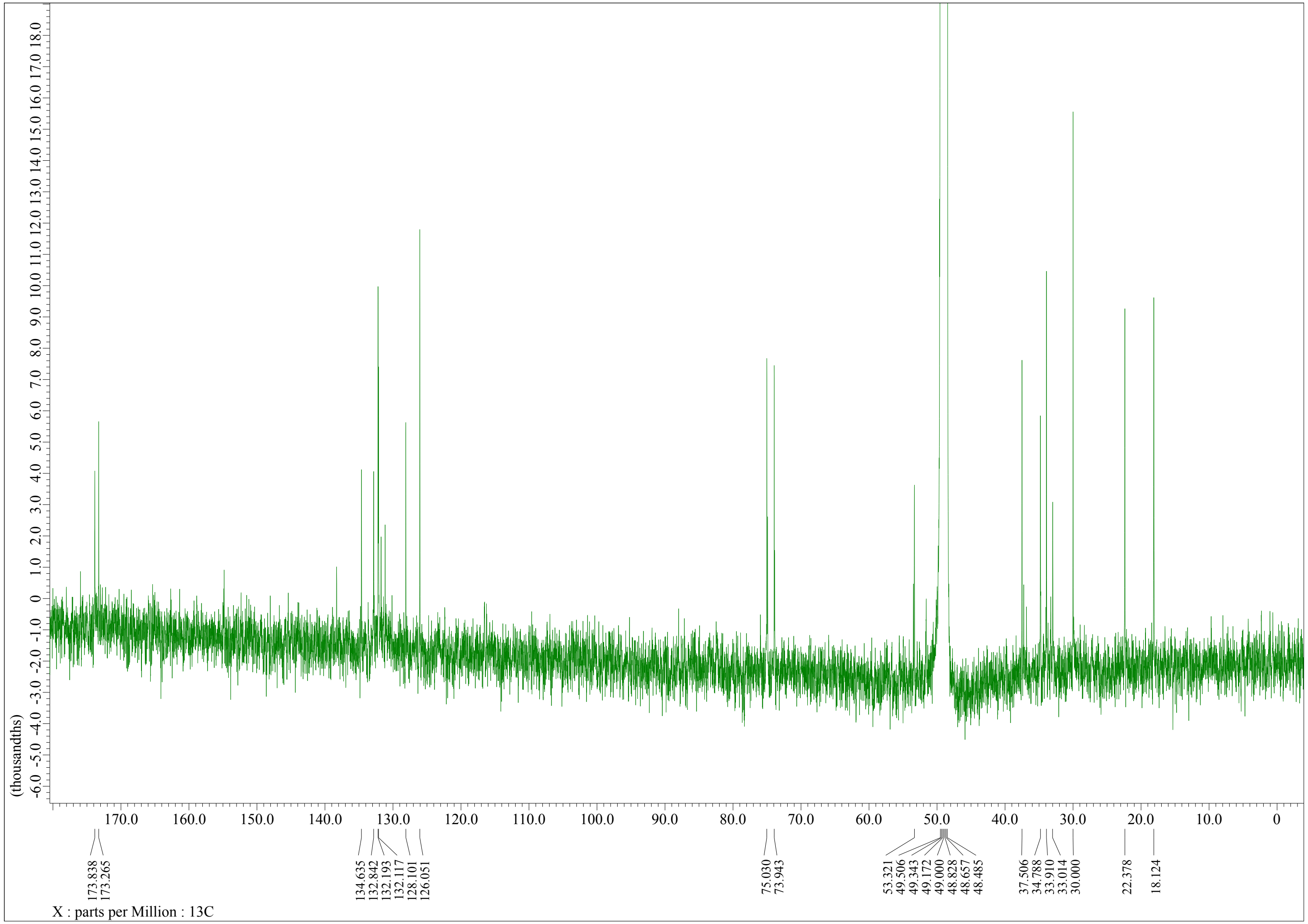

Figure S8 ^{13}C NMR (125 MHz, CD_3OD) spectrum of **2**

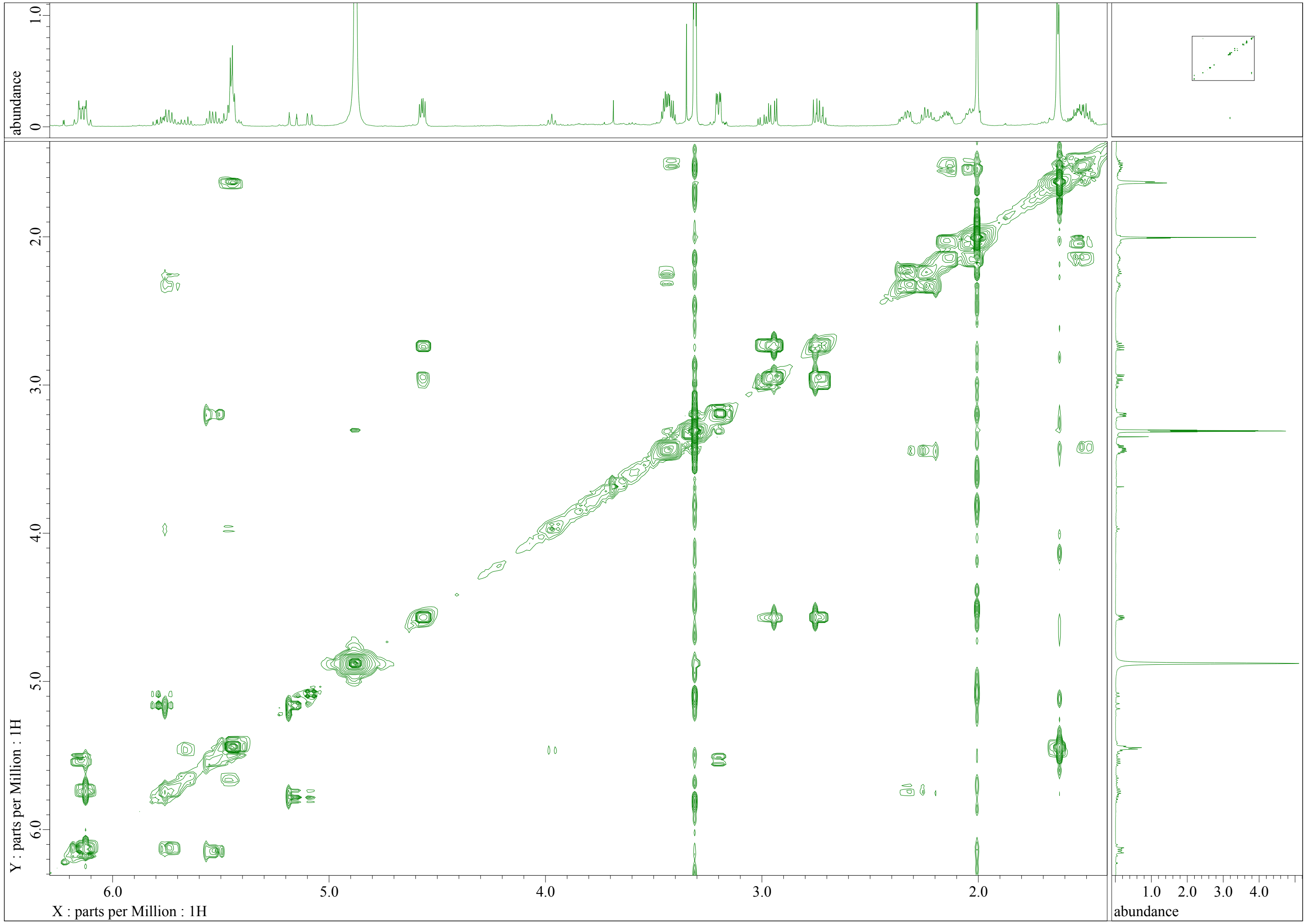

Figure S9 ^{1}H–^{1}H COSY (CD_3OD) spectrum of **2**

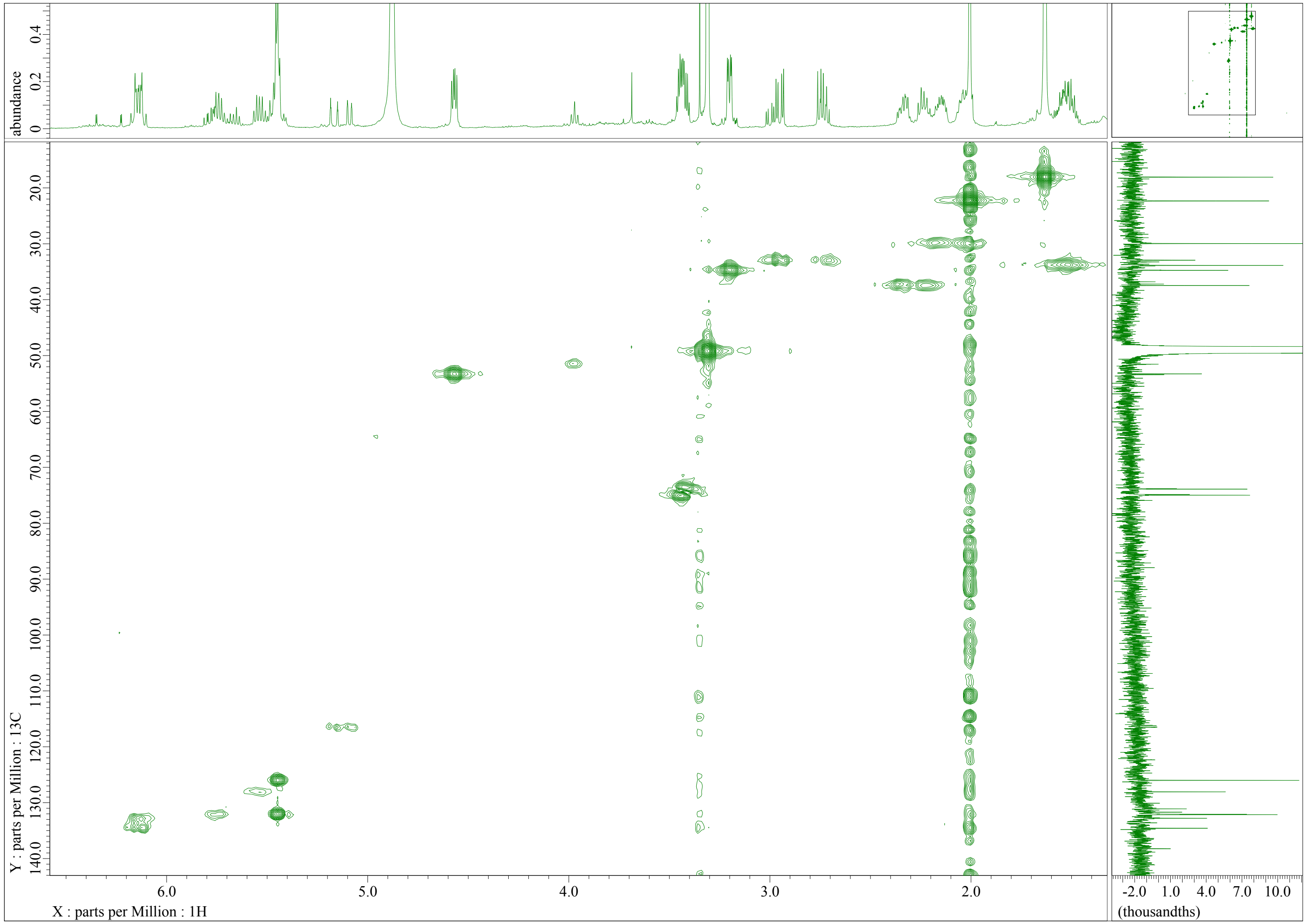

Figure S10 HMQC (CD_3OD) spectrum of **2**

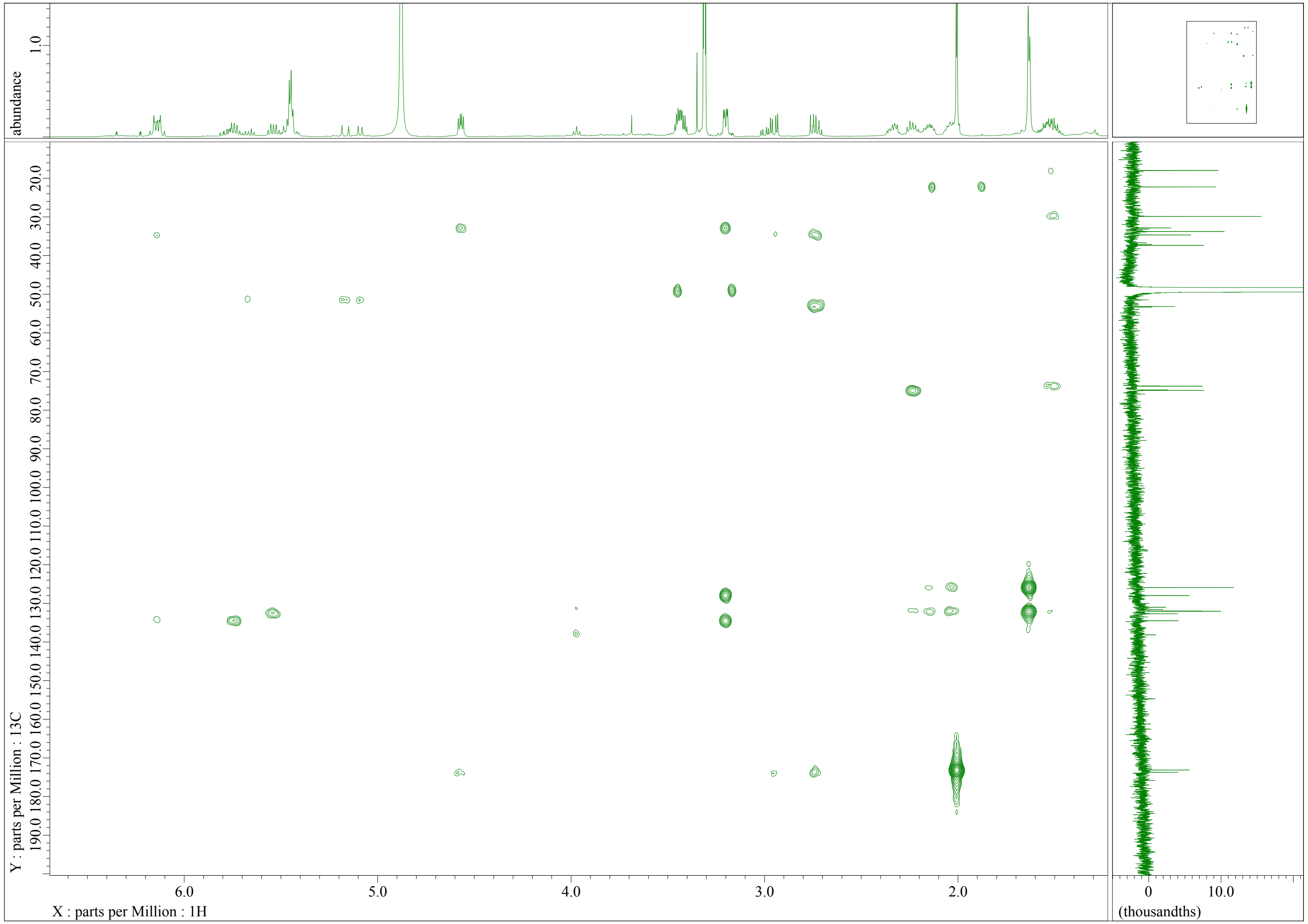

Figure S11 HMBC (CD_3OD) spectrum of **2**

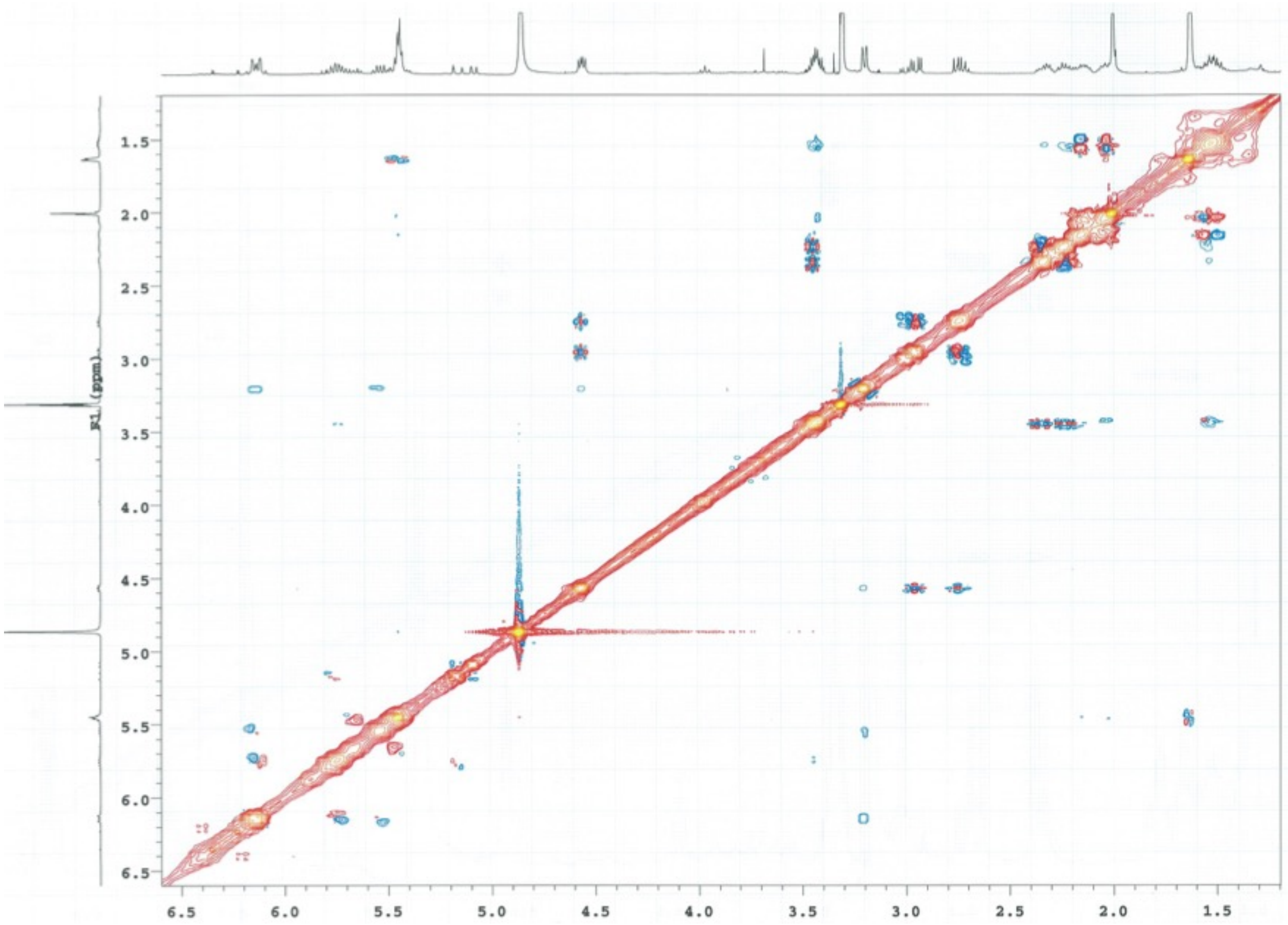

Figure S12 ROESY (CD_3OD) spectrum of **2**

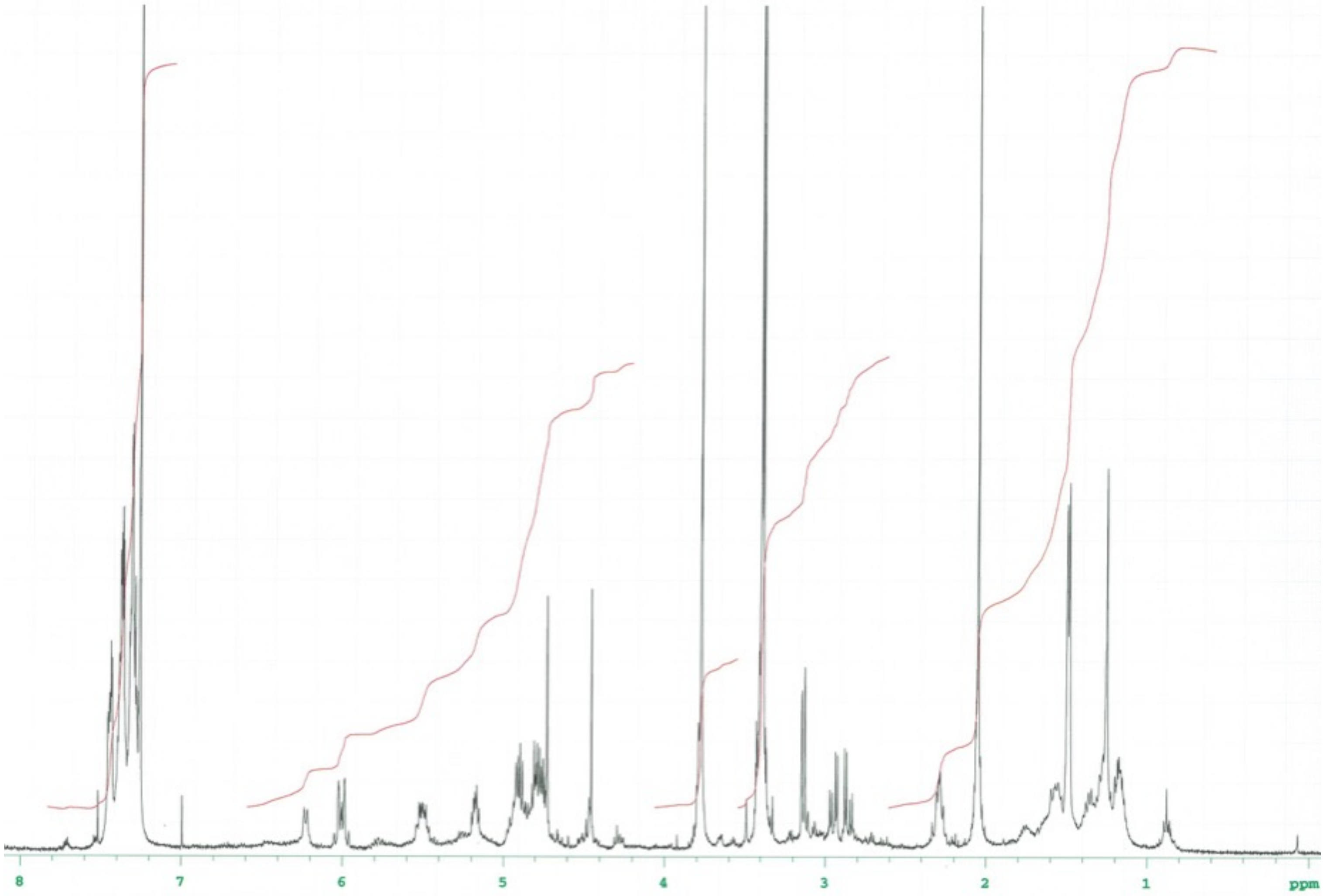

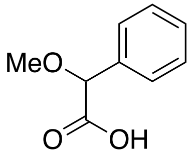

MPA

Figure S13 1H-NMR (600 MHz, $CDCl_3$) spectrum of **4** measured at 25°C and the structure of MPA.

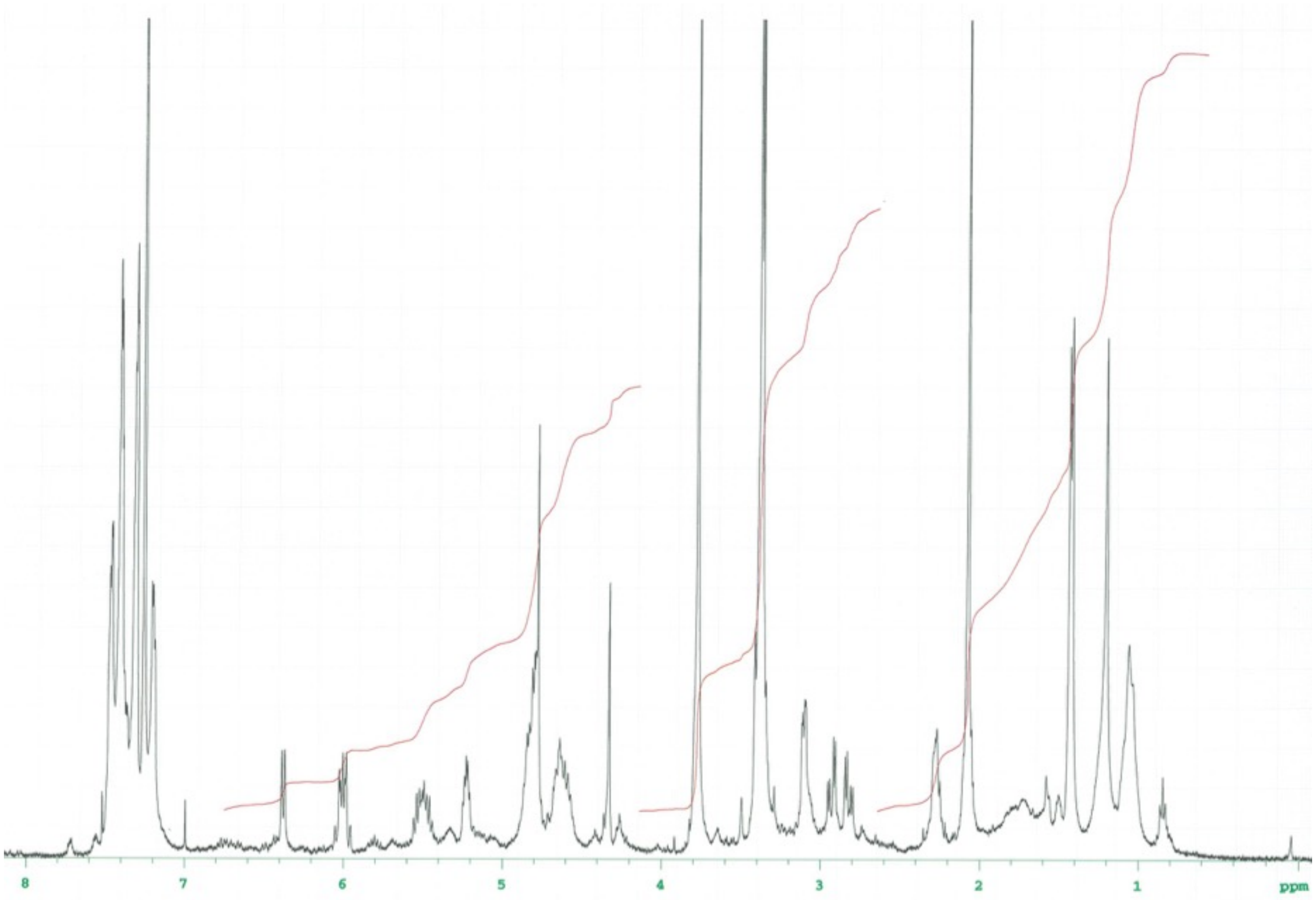

Figure S14 ^{1}H NMR (500 MHz, $CDCl_3$) spectrum of **4** measured at –30°C.
